# Panthenol Protects Against Oxidative Stress and Liver Fibrosis in Cholestasis in Association with Increased Coenzyme A Biosynthesis

**DOI:** 10.3390/ijms27114913

**Published:** 2026-05-29

**Authors:** Dmitry S. Semenovich, Polina A. Abramicheva, Ljubava D. Zorova, Andrey V. Elchaninov, Olga V. Markova, Nadezda V. Andrianova, Valentina A. Babenko, Nina P. Kanunnikova, Andrey G. Moiseenok, Irina B. Pevzner, Marina I. Buyan, Egor Y. Plotnikov, Dmitry B. Zorov

**Affiliations:** 1 A.N. Belozersky Institute of Physico-Chemical Biology, M.V. Lomonosov Moscow State University, 119992 Moscow, Russia; abramicheva.polina@belozersky.msu.ru (P.A.A.); ljuzor@belozersky.msu.ru (L.D.Z.); markova@belozersky.msu.ru (O.V.M.); andrianova@belozersky.msu.ru (N.V.A.); babenval90@gmail.com (V.A.B.); pevzner_ib@belozersky.msu.ru (I.B.P.); marinanenart@gmail.com (M.I.B.); zorov@belozersky.msu.ru (D.B.Z.); 2 V.I. Kulakov National Medical Research Center for Obstetrics, Gynecology and Perinatology, 117997 Moscow, Russia; 3 Avtsyn Research Institute of Human Morphology, Federal State Budgetary Scientific Institution “Petrovsky National Research Centre of Surgery”, 117418 Moscow, Russia; elchandrey@yandex.ru; 4 Department of Technology, Physiology and Food Hygiene, Yanka Kupala State University of Grodno, 230030 Grodno, Belarus; n.kanunnikova@grsu.by; 5 Institute of Biochemistry of Biologically Active Compounds, 230030 Grodno, Belarus; andrey.moiseenok@tut.by

**Keywords:** alcohol dehydrogenase, bile duct ligation, coenzyme A, hepatic stellate cells, hopantenic acid, glutathione system, lipid peroxidation, liver fibrosis, mitochondrial dysfunction, oxidative stress, panthenol, pantethine

## Abstract

We explored the possibility of antioxidant and antifibrotic effects of panthenol (PL) associated with modulation of coenzyme A (CoA) biosynthesis in the liver in a rat model of chronic obstructive cholestasis induced by bile duct ligation (BDL). We found that PL increased alcohol dehydrogenase (ADH) activity in the liver of BDL rats. PL and its analog pantethine increased pantothenate kinase (PANK) activity, restored hepatic CoA levels reduced by BDL, lowered protein-bound CoA, and normalized impaired mitochondrial functions associated with induced oxidative stress after BDL. These effects were accompanied by decreased collagen deposition and improved morphological features of hepatocytes. In contrast, PANK inhibitor, hopantenic acid (HPA), reduced hepatic CoA levels, aggravated hepatocellular damage, and promoted fibrosis. In the human hepatic stellate cell line LX-2, PL exhibited no cytotoxicity over a wide concentration range, increased intracellular CoA levels, decreased reactive oxygen species (ROS) production, and attenuated collagen accumulation associated with oxidative stress in vitro. Importantly, inhibition of ADH by 4-methylpyrazole completely abolished the protective effects of panthenol, indicating that its activity depends on metabolic pathways involving CoA. Notably, PL did not directly reduce H_2_O_2_ or superoxide anion radical production in cell-free systems but significantly suppressed lipid peroxidation in liposomes and red blood cells in vitro. Ultimately, these findings indicate that the antioxidant and antifibrotic effects of PL are associated with modulation of CoA metabolism and enhanced resistance of biological membranes to oxidative damage.

## 1. Introduction

Liver fibrogenesis is a dynamic and highly integrated tissue, cellular, and molecular process that, during chronic liver disease (CLD), gradually leads to excessive deposition of extracellular matrix components, possibly aimed to diminish negative consequences [1]. Fibrosis develops in response to chronic damage to liver cells caused by various factors, such as ethanol consumption, viral attack, exposure to toxins and drugs, or cholestasis [2]. Chronic stimuli involved in the initiation of fibrosis lead to oxidative stress and the formation of reactive oxygen species (ROS), which act as mediators of molecular events involved in the pathogenesis of liver fibrosis [3].

Oxidative stress is a key process leading to liver injury and initiation of liver fibrosis, characterized by distortion of redox balance in liver cells and accompanied by intensive generation of free radicals. ROS belong to a family of profibrotic mediators, and include superoxide anion radicals, hydrogen peroxide (H_2_O_2_), and hydroxyl radicals [4]. They are excessively produced in CLD during electron transport and lipid peroxidation in mitochondria, hepatocytes, hepatic stellate cells (HSC), and macrophages. It is known that lipid peroxidation in hepatocytes, as well as ROS generated in neutrophils and CYP2E1, contribute to the expression of type I collagen in HSC [5]. In this context, the use of redox-modulating natural and synthetic compounds remains a relevant approach for modulating redox status and reducing oxidative stress in liver cells as antifibrotic therapy in biomedicine dealing with CLD.

Panthenol (PL) is an alcohol derivative of pantothenic acid (vitamin B5). PL exhibits anti-inflammatory, antiapoptotic, and antioxidant activity and has been successfully used as a hepatoprotective agent in in vivo models of acute subtotal hepatic ischemia–reperfusion, toxic liver injury induced by cisplatin and methotrexate, and sepsis [6,7]. However, to date, PL has not been used in chronic models of CLD for antifibrotic therapy. The mechanism of its antioxidant and antifibrotic action in liver injury remains unclear. It has been previously shown that pantothenic acid effectively prevents fibrosis in a thioacetamide-induced model of liver injury in mice [8]. Thus, PL, being a derivative of pantothenic acid, may be considered a potential compound for use in antifibrotic therapy of CLD.

PL, like pantothenic acid, serves as a substrate for the biosynthesis of coenzyme A (CoA), which has recently been shown to exhibit antioxidant properties [9]. It has been demonstrated that CoA can reversibly bind to free sulfhydryl groups of cysteine residues in proteins under conditions of oxidative stress, thereby protecting them from irreversible oxidation [10,11]. An important observation is that mitochondria of liver cells contain comparable levels of CoA and reduced glutathione (GSH) in the matrix (approximately 5 mM), suggesting the potential role of CoA in maintaining thiol redox balance in mitochondria [12,13]. This is supported by similar redox potentials of the CoA/CoA disulfide (−234 mV) and GSH/glutathione disulfide (−240 mV) pairs at pH 7.0 [14]. Therefore, mitochondria may represent a key target for regulation of thiol redox balance and prevention of oxidative stress. We hypothesized that this state can be achieved by stabilizing redox potential through administration of PL as a precursor of CoA biosynthesis. It is known that precursors of GSH biosynthesis, such as S-adenosylmethionine (SAM) and N-acetylcysteine (NAC), are widely used to maintain redox balance and protect liver cells from injury and fibrosis [15,16,17]. We assumed that a similar approach could be applied to the CoA system by enhancing its antioxidant potential through administration of its biosynthetic precursors, such as PL or pantethine (PT). Conversely, inhibition of key enzymes of CoA biosynthesis, for example, by hopantenic acid (HPA, N-pantoyl-GABA), a structural analog of pantothenic acid and inhibitor of pantothenate kinase (PANK) [18], may lead to decreased thiol redox status and increased susceptibility of cells to damage. At present, experimental approaches aimed at redox modulation of CoA levels and protein CoAlation to enhance cellular protection against oxidative stress remain limited, which highlights the relevance of this research direction.

The aim of the present study was to investigate the mechanisms underlying the antioxidant and antifibrotic effects of PL, with particular emphasis on its association with the CoA biosynthesis system and regulation of thiol redox status in a model of chronic obstructive cholestasis in rats and oxidative stress in vitro.

## 2. Results

### 2.1. Evaluation of the Hepatoprotective Effect of Panthenol as a Modulator of Redox Status and CoA Biosynthesis in a Model of Chronic Obstructive Cholestasis in Rats

In the first part of the study, we evaluated changes in oxidative stress parameters associated with mitochondrial dysfunction and progression of liver fibrosis in rats with chronic obstructive cholestasis following administration of PL and other alternative modulators of CoA biosynthesis (PT and HPA). PT and HPA were used as alternative pharmacological modulators of the CoA system, serving as positive and negative controls, respectively. PT, the disulfide form of pantetheine and a native CoA precursor, can serve as a substrate for CoA biosynthesis under physiological conditions. HPA was used in our model as an available pharmacological inhibitor of CoA biosynthesis, reported to inhibit PANK, the first enzyme of the CoA biosynthetic pathway. To assess the relationships between oxidative stress, fibrosis, and the CoA system, we measured total, free, and protein-bound CoA levels and determined the activity of key enzymes involved in the metabolism of the studied compounds. In addition to evaluating the efficiency of hepatoprotective therapy with CoA biosynthesis modulators (PL, PT, and HPA), histopathological analysis of liver tissue sections was performed.

Chronic obstructive cholestasis was induced by BDL in rats (see Section 4.2). Administration of PL and other CoA modulators (PT and HPA) was initiated 3 weeks after BDL, at a stage when stable signs of obstructive jaundice and moderate morphological changes associated with inflammation, oxidative stress, and early fibrosis had already developed [19]. The compounds were administered daily by intragastric gavage at a dose of 500 mg/kg from weeks 3 to 6 of the experiment. These doses correspond to an equivalent amount of approximately 2 mmol/kg in terms of pantothenic acid. Rats were euthanized at week 6, and liver tissue was collected for biochemical and histological analyses.

**Figure 1:**
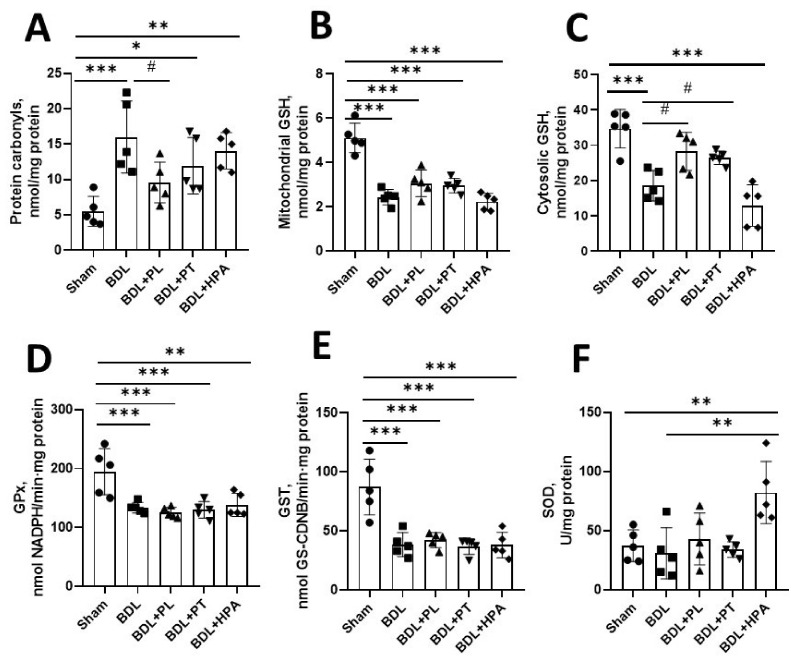
Assessment of oxidative stress parameters, glutathione levels, and the activity of glutathione-metabolizing enzymes and SOD in the liver of rats with chronic obstructive cholestasis (BDL, 6 weeks) treated with PL, PT, and HPA (from weeks 3 to 6 after BDL). (**A**) Mitochondrial protein carbonyl levels; (**B**) Mitochondrial GSH levels; (**C**) Cytosolic GSH levels; (**D**) Mitochondrial glutathione peroxidase (GPx) activity; (**E**) Mitochondrial glutathione S-transferase (GST) activity; (**F**) Mitochondrial SOD activity. * *p* < 0.05, ** *p* < 0.01, *** *p* < 0.001 vs. control group; # *p* < 0.05 vs. BDL group (ANOVA with Dunnett’s test). Each dot in the graph represents the individual value of the measured parameter.

Chronic obstructive cholestasis in rats was associated with pronounced oxidative modifications in the liver, manifested by a threefold increase in mitochondrial protein carbonyls and more than a twofold decrease in mitochondrial GSH levels (Figure 1A,B). In the presence of PL, the increase in mitochondrial protein carbonyls was significantly lower compared to untreated BDL rats, whereas in PT and HPA-treated groups, this parameter remained elevated, similar to the BDL group. It should be noted that administration of PL, PT, and HPA did not restore mitochondrial GSH levels (Figure 1B). In contrast, cytosolic GSH levels increased approximately 1.5-fold in PL and PT-treated rats, but not in the HPA group, compared to BDL animals.

**Figure A1:**
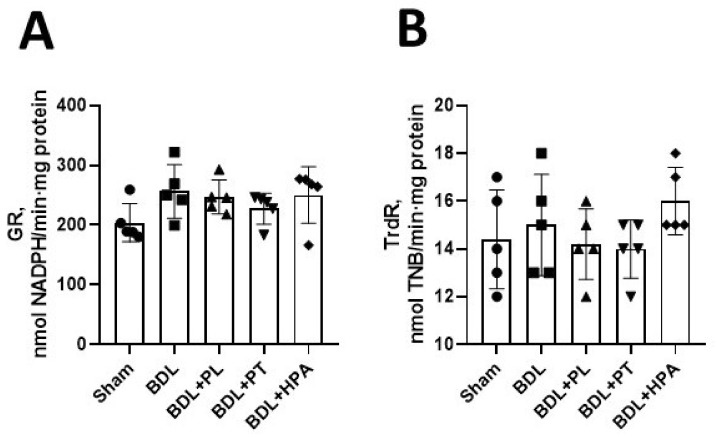
Mitochondrial glutathione reductase and thioredoxin reductase activities in rat liver following BDL (6 weeks) and treatment with panthenol and other CoA biosynthesis modulators (weeks 3–6 after BDL). (**A**) Mitochondrial glutathione reductase (GR) activity; (**B**) Mitochondrial thioredoxin reductase (TrdR) activities. Each dot in the graph represents the individual value of the measured parameter.

The observed depletion of mitochondrial GSH may be related to its excessive oxidation, increased conjugation with hepatic metabolites, and/or impaired transport of GSH from the cytosol into mitochondria, since the activity of mitochondrial GSH-metabolizing enzymes—glutathione peroxidase (GPx) and glutathione-S-transferase (GST)—was markedly decreased in all BDL groups, regardless of treatment (Figure 1D,E). Importantly, under conditions of hepatic oxidative stress, mitochondrial glutathione reductase (GR) and thioredoxin reductase (TrdR) activities remained unchanged in all BDL groups (see Appendix A, Figure A1). It is possible that depletion of the mitochondrial GSH pool is associated with enhanced non-enzymatic oxidation by ROS. Mitochondrial superoxide dismutase (SOD) activity increased significantly only in HPA-treated rats (Figure 1F). Notably, HPA administration in BDL rats was characterized by a more pronounced decrease in cytosolic GSH and a greater increase in mitochondrial SOD activity compared to the untreated BDL group, which was not observed in PL- and PT-treated animals.

**Figure 2:**
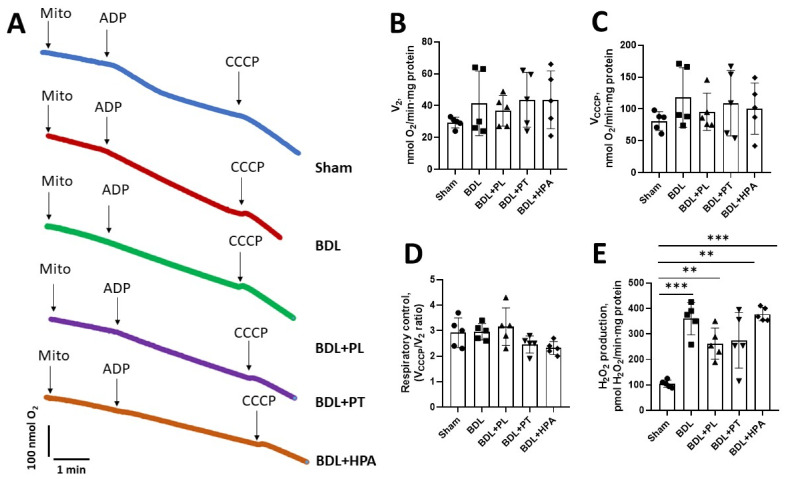
Assessment of mitochondrial respiratory activity and H_2_O_2_ production rates in isolated liver mitochondria from rats with chronic obstructive cholestasis (BDL, 6 weeks) treated with PL, PT, and HPA (from weeks 3 to 6 after BDL). (**A**) Representative polarographic traces showing oxygen consumption during succinate oxidation (State 2) and after the addition of ADP (State 3) and CCCP (uncoupled respiration); (**B**) Respiration rate in State 2; (**C**) Respiration rate after CCCP addition; (**D**) Respiratory control ratio (V_CCCP_/V_2_). (**E**) Rate of H_2_O_2_ production by mitochondria. ** *p* < 0.01, *** *p* < 0.001 (ANOVA with Dunnett’s test). Each dot in the graph represents the individual value of the measured parameter.

Alterations in redox balance and the development of oxidative stress in liver mitochondria of BDL rats were accompanied by pronounced changes in mitochondrial respiratory activity (Figure 2A). Under BDL conditions, a tendency toward an increase in the rate of succinate-dependent respiration in State 2 was observed (Figure 2B), as well as an increase in maximal oxygen consumption following the addition of the uncoupler CCCP (Figure 2C). The absence of statistically significant changes in the respiratory control ratio (Figure 2D) indicates preserved mitochondrial coupling and may reflect a compensatory adaptive rearrangement of mitochondrial respiration in the liver of BDL rats.

An important feature was the lack of transition of mitochondria isolated from BDL rats to the active ADP-stimulated State 3, as well as the absence of the classical increase in oxygen consumption following uncoupler addition. Notably, PL and PT, to a greater extent than HPA, prevented these alterations. In addition, more than a threefold increase in H_2_O_2_ production by liver mitochondria from BDL rats during succinate oxidation was observed. Administration of PL and PT, but not HPA, tended to reduce this parameter (Figure 2E).

**Figure 3:**
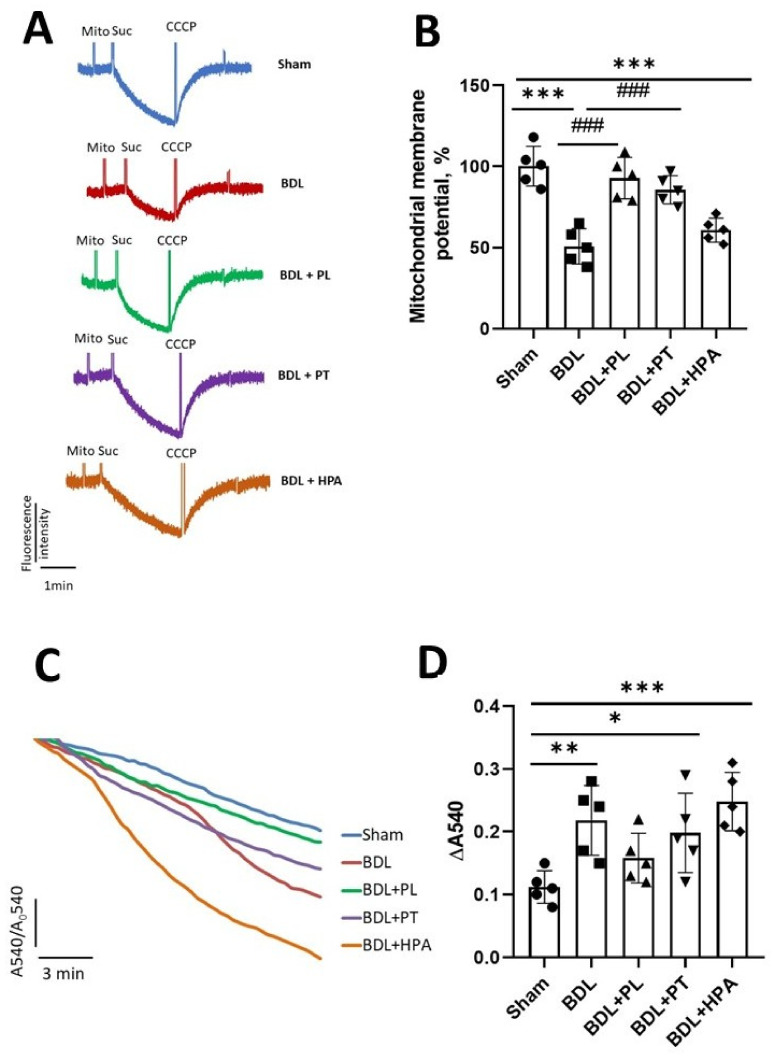
Assessment of mitochondrial membrane potential and Ca^2+^-induced swelling in isolated liver mitochondria from rats with chronic obstructive cholestasis (BDL, 6 weeks) treated with PL, PT, and HPA (from weeks 3 to 6 after BDL). (**A**) Representative kinetic traces showing changes in mitochondrial membrane potential upon energization with succinate in the presence of rotenone and subsequent depolarization induced by CCCP; (**B**) Quantification of mitochondrial membrane potential expressed as a percentage relative to the control group. (**C**) Representative traces of Ca^2+^-induced mitochondrial swelling. (**D**) Quantification of Ca^2+^-induced mitochondrial swelling based on changes in light scattering amplitude at 540 nm. * *p* < 0.05, ** *p* < 0.01, *** *p* < 0.001 vs. control group; ### *p* < 0.001 vs. BDL group (ANOVA with Dunnett’s test). Each dot in the graph represents the individual value of the measured parameter.

Under obstructive cholestasis, liver mitochondria of rats were in a partially depolarized state, as evidenced by a twofold decrease in mitochondrial membrane potential and a reduced signal amplitude upon CCCP addition to energized mitochondria in a medium containing succinate in the presence of rotenone (Figure 3A,B). Administration of PL and PT, but not HPA, to BDL rats resulted in a significant restoration of mitochondrial membrane potential to values close to those of the control group.

In addition, liver mitochondria from BDL rats were characterized by increased sensitivity to Ca^2+^-induced swelling associated with the opening of the mitochondrial permeability transition pore (mPTP) (Figure 3C,D). PL, to a greater extent than PT, reduced the amplitude of mitochondrial swelling, whereas HPA further increased mitochondrial swelling in the liver of BDL rats.

**Figure 4:**
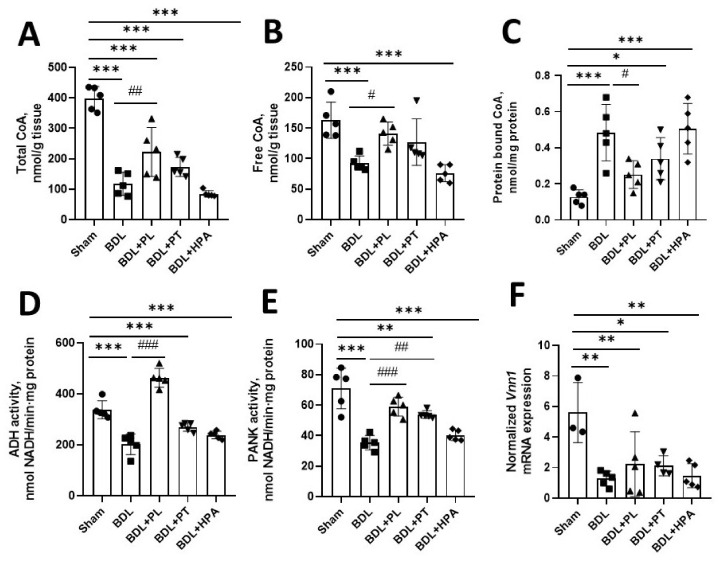
Coenzyme A metabolism in the liver of rats with chronic obstructive cholestasis (6-week BDL) and treatment with CoA biosynthesis modulators (PL, PT, and HPA from week 3 to week 6 after BDL). (**A**) Total CoA content; (**B**) Free CoA content; (**C**) Protein-bound CoA level; (**D**) Alcohol dehydrogenase activity (ADH); (**E**) Pantothenate kinase activity (PANK); (**F**) Relative mRNA expression of *Vnn1*, normalized to *Rplp0*. * *p* < 0.05, ** *p* < 0.01, *** *p* < 0.001 vs. control group; # *p* < 0.05, ## *p* < 0.01, ### *p* < 0.001 vs. BDL group (one-way ANOVA, Dunnett’s test). Each dot in the graph represents the individual value of the measured parameter.

Taken together, the observed changes in liver mitochondria indicate pronounced oxidative stress, manifested by a marked suppression of the GSH system and impairment of its functional activity, suggesting profound metabolic remodeling in the liver under cholestatic hepatocellular injury induced by BDL. To further elucidate the mechanisms underlying these changes, we analyzed the CoA biosynthesis pathway in the liver, with particular attention to total, free, and protein-bound CoA levels, as well as the activity and expression of key enzymes involved in the biotransformation of PL and other CoA system modulators, PT and HPA (Figure 4).

In BDL rats, a fourfold decrease in total CoA content and a 1.5-fold decrease in free CoA levels were observed in liver tissue compared to the control group. Administration of PL to BDL rats effectively restored total CoA levels in liver tissue approximately twofold and free CoA levels by 1.5-fold; however, CoA levels were not fully restored to those of the control group (Figure 4A,B). Administration of PT to BDL rats resulted in a similar tendency toward normalization of CoA levels, whereas HPA (a competitive PANK inhibitor) did not demonstrate a restorative effect, and CoA levels remained significantly reduced.

In contrast, protein-bound CoA levels in liver tissue were markedly increased in BDL rats, indicating enhanced conjugation of CoA with proteins under oxidative stress conditions. Only PL treatment significantly reduced this parameter by more than 1.5-fold, approaching but not reaching control levels. PT showed a similar tendency to reduce protein-bound CoA, whereas HPA had no effect (Figure 4C). The measured protein-bound CoA fraction is an operational fraction obtained after protein precipitation and reductive extraction. Therefore, in addition to CoA released from protein mixed disulfides, this fraction may also include a minor contribution from precipitated acyl-CoA species. The sum of panels B and C should not be interpreted as quantitatively identical to the total CoA in panel A. We suggest that under chronic cholestasis, oxidative stress may promote the formation of protein-bound CoA (and/or GSH conjugates), which may serve as a reversible reservoir of cellular thiols and protect proteins from irreversible oxidative damage caused by ROS.

Administration of PL to BDL rats, in contrast to PT and HPA, resulted in induction of alcohol dehydrogenase (ADH) activity, with an increase of more than 1.4-fold compared to the BDL group (Figure 4D). This effect is supported by the increase in CoA levels, which are likely derived from PL via the classical metabolic pathway through pantothenate, a product of the ADH-mediated reaction. On the other hand, further biotransformation of pantothenate is associated with changes in PANK activity. Administration of PL and PT, but not HPA, was accompanied by an approximately 1.5-fold increase in PANK activity in the liver of BDL rats, indicating activation of CoA biosynthesis (Figure 4E).

To evaluate a potential alternative endogenous source of hepatic pantothenate, we assessed the mRNA expression of vanin-1 (*Vnn1*, pantetheinase). In BDL rats, *Vnn1* expression was decreased threefold, whereas treatment with PL, PT, or HPA did not restore its expression, suggesting that the pantetheine hydrolysis pathway does not significantly contribute to modulation of hepatic CoA levels under cholestatic conditions (Figure 4F).

Thus, chronic obstructive cholestasis induced by BDL leads to a pronounced suppression of CoA biosynthesis in the liver and a shift in its metabolic balance toward protein-bound mixed disulfide forms, reflecting processes of protein CoAlation and oxidative stress. Among the studied modulators of CoA biosynthesis, PL demonstrated the most pronounced ability to restore CoA levels by increasing the free CoA fraction and reducing protein-bound CoA, whereas PT showed a weaker effect compared to PL, and HPA treatment did not show any improvement.

**Figure 5:**
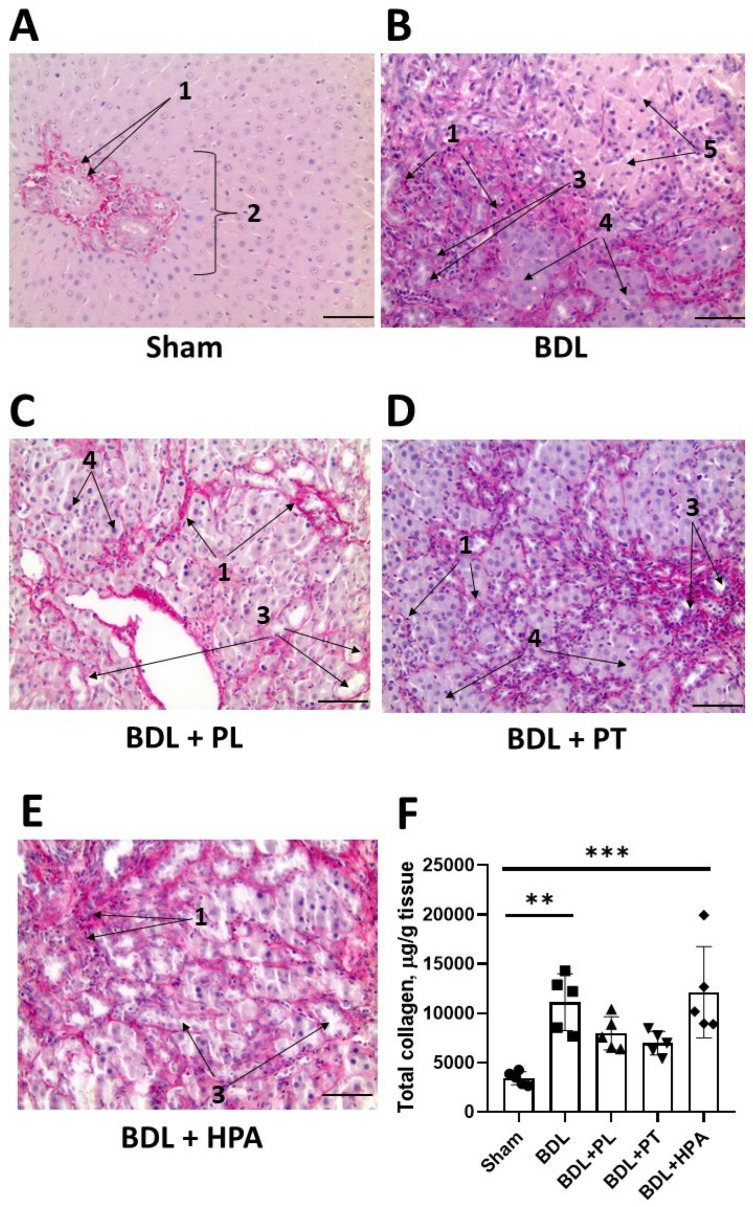
Representative liver sections stained with Sirius Red. (**A**) Normal liver architecture in sham-operated rats after 6 weeks of laparotomy. (**B**–**E**) Histological alterations after BDL and administration of CoA modulators (PL, PT, and HPA from week 3 to week 6 after BDL). The labels on the micrograph correspond to the following structures in the legend: 1—collagen fibers; 2—portal triad; 3—bile ducts; 4—hypertrophic hepatocytes; 5—necrosis. (**F**) Total collagen content in liver tissue. ** *p* < 0.01, *** *p* < 0.001 (one-way ANOVA, Dunnett’s test). Arrows and brackets indicate the corresponding structures. Bar, 100 µm. Each dot in panel (**F**) represents the individual value of the measured parameter.

We also evaluated the antifibrotic and hepatoprotective effects of PL and other CoA biosynthesis modulators (PT and HPA) by microscopic examination of liver tissue sections stained with Sirius Red (Figure 5). In sham-operated rats, the liver parenchyma exhibited a typical structure consisting of classical hexagonal hepatic lobules (Figure 5A). Hepatocytes formed trabeculae, between which sinusoidal capillaries were located, converging toward the center of the hepatic lobule. Central veins were located in the middle of the lobules. Portal triads (indicated by brackets), consisting of an interlobular vein, artery, and bile duct, were located at the periphery of the lobules. The boundaries between lobules were poorly distinguishable due to the small amount of connective tissue.

Six weeks after BDL, the structure of the liver parenchyma was markedly altered (Figure 5B). A pronounced proliferation of interlobular connective tissue was observed. Numerous interlobular bile ducts were present within the fibrous tissue, accompanied by inflammatory infiltration, hypertrophy of some hepatocytes, and progressive bile duct hyperplasia.

Administration of PL to BDL rats reduced fibrosis progression, with PL being more effective than PT. In PL-treated animals, a decrease in the number of interlobular bile ducts (indicated by arrows) was observed, inflammatory infiltration was nearly absent, areas of necrosis were reduced, and isolated hypertrophied hepatocytes were detected (Figure 5C). In contrast, PT treatment was associated with persistent enlargement of interlobular bile ducts (indicated by arrows), typical of BDL animals, although this effect was less pronounced compared to PL treatment (Figure 5D). In addition, rats in the BDL + PT group exhibited more pronounced inflammatory infiltration and connective tissue proliferation, as well as the presence of hypertrophied hepatocytes and necrotic areas, compared to the BDL + PL group.

Administration of HPA to BDL rats exacerbated liver injury and fibrosis progression (Figure 5E). In the BDL + HPA group, histological changes were similar to those observed in the BDL group. Notably, in the BDL + HPA group, proliferation of connective tissue and interlobular bile ducts was more pronounced than in the BDL group, as was inflammatory infiltration in the bile duct regions.

We also summarized the histopathological changes detected in the liver after BDL-induced injury and treatment with PL and alternative modulators of the CoA system (PT and HPA). For this purpose, an integrated pathological score was calculated as the sum of scores for four pathological parameters: inflammation, necrosis, ductular reaction, and fibrosis. Each parameter was assessed using a 0–3 scoring scale.

As shown in Table 1, BDL-induced liver injury in rats was characterized by the highest integrated score. Administration of PL, and to a lesser extent PT, reduced histopathological alterations in the liver of BDL rats. In contrast, HPA did not exert a hepatoprotective effect and aggravated the severity of liver injury in BDL rats.

**Figure 6:**
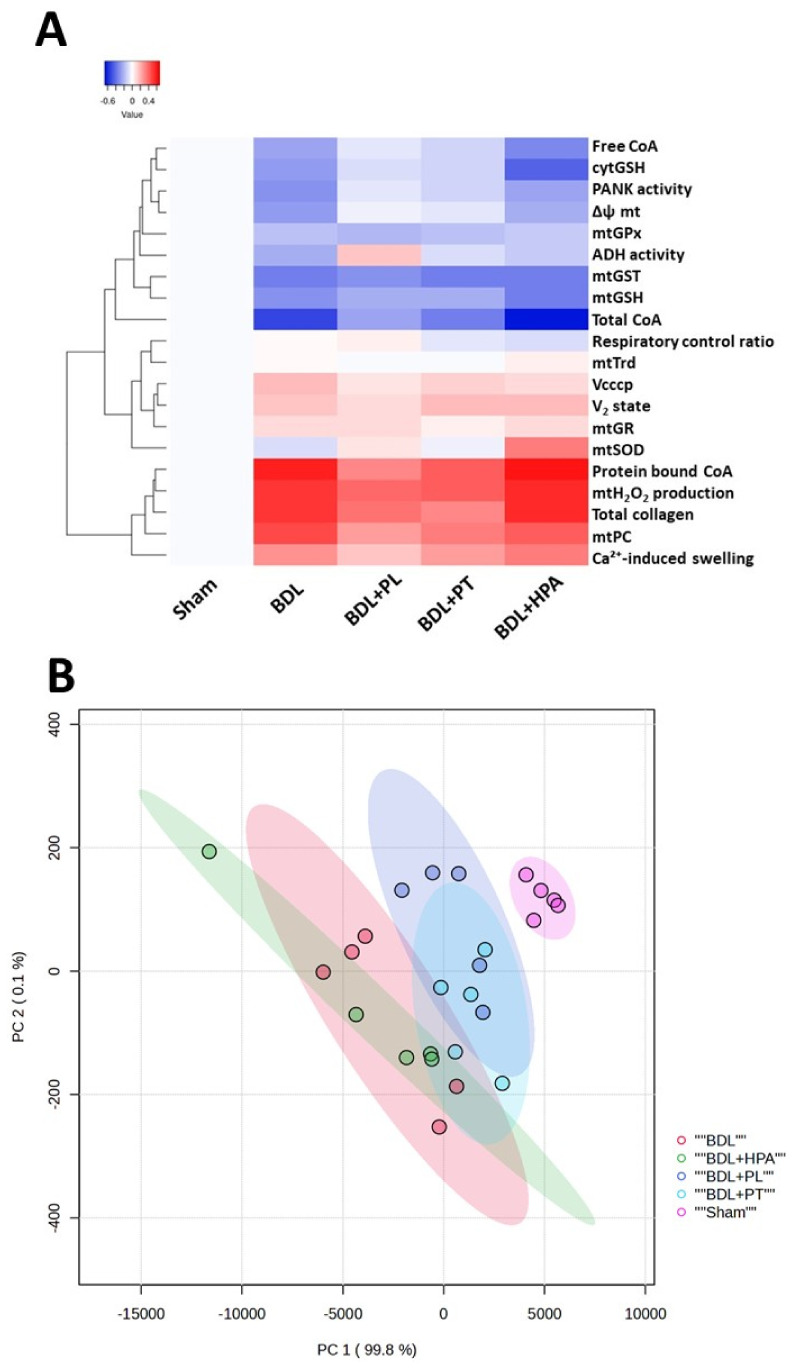
Effect of PL, PT, and HPA treatment in rats with 6-week BDL on the changes in mitochondrial functional activity and biochemical parameters of oxidative stress, glutathione, and CoA systems in the liver. Obstructive cholestasis treatment with CoA biosynthesis modulators (PL, PT, and HPA) was carried out from week 3 to week 6 after BDL. (**A**) Cluster analysis and heat map to evaluate the systemic changes in the liver after bile duct ligation treatment with CoA biosynthesis modulators. Where “mt” denotes mitochondrial parameters, “cyt” refers to the liver cytosol, “Δψ” indicates mitochondrial membrane potential, and “PC” represents protein carbonyls. (**B**) Principal component analysis visualization to evaluate the liver metabolic changes after bile duct ligation treatment with CoA biosynthesis modulators.

As shown in Figure 6A, parameters of the CoA biosynthesis system and the GSH system are grouped into a single cluster (blue), whereas the lower cluster (red) combines parameters related to oxidative metabolism under conditions of chronic obstructive cholestasis. PCA supports an association between mitochondrial dysfunction, redox balance, and CoA system alterations, which play a significant role in the regulation of oxidative damage in BDL, thereby contributing to the progression and severity of fibrosis.

Figure 6B illustrates the identified systemic metabolic changes through visualization of data distribution obtained by principal component (PC) analysis. PL and PT, as precursors and substrates of CoA biosynthesis, form closely related clusters, in contrast to HPA, which supports our hypothesis regarding the potential role of CoA system modulation in hepatoprotection.

Thus, PL and PT can be characterized as the most effective compounds in modulating redox status and mitochondrial energy metabolism in liver cells of BDL rats, reducing bile duct hyperplasia and slowing the progression of liver fibrosis. Notably, PL treatment was more effective than PT, which may be associated with differences in the activity of enzymes involved in their biotransformation. In contrast, HPA, as an inhibitor of CoA biosynthesis, exacerbated fibrotic changes, maintained persistent bile duct hyperplasia, and worsened liver parenchymal injury.

Since treatment was initiated at 3 weeks after BDL, the therapeutic effects of PL and PT were relatively moderate, likely due to the persistence of the underlying mechanical obstruction, which drives obstructive jaundice, sustained oxidative stress, and liver fibrosis.

### 2.2. CoA-Dependent Mechanisms of the Antifibrotic and Antioxidant Effects of Panthenol in Human Hepatic Stellate Cells (LX-2) Under Oxidative Stress In Vitro

To elucidate the CoA-mediated pathway underlying the protective effects of PL under conditions of oxidative stress and fibrosis, additional in vitro studies were performed using the human hepatic stellate cell line (LX-2). This cell model is widely used in biomedical research for evaluating the antifibrotic activity of various chemical and natural compounds, as demonstrated in previous studies [20,21].

**Figure 7:**
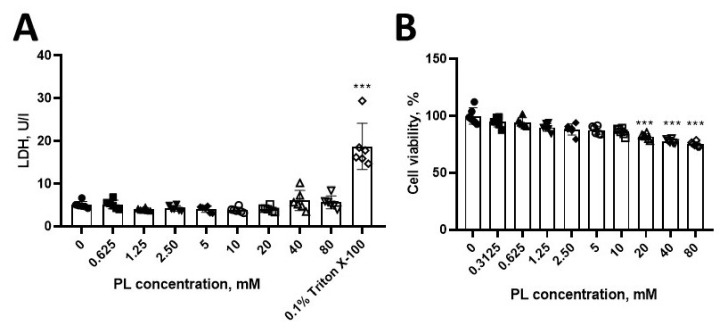
Assessment of cytotoxicity and cell viability of LX-2 cells after 24 h exposure to different concentrations of PL. (**A**) LDH activity in the culture medium; (**B**) MTT assay. *** *p* < 0.001 vs. control (0 mM panthenol) (one-way ANOVA with Dunnett’s test).

After 24 h incubation of LX-2 cells with PL at concentrations ranging from 0.625 to 80 mM in DMEM/F-12 medium supplemented with 10% fetal bovine serum, no increase in lactate dehydrogenase (LDH) activity in the culture medium was observed, indicating the absence of cytotoxic effects of PL (Figure 7A). Only at PL concentrations above 10 mM was a moderate decrease in cell viability (18–20%) detected using the MTT assay (Figure 7B).

**Figure 8:**
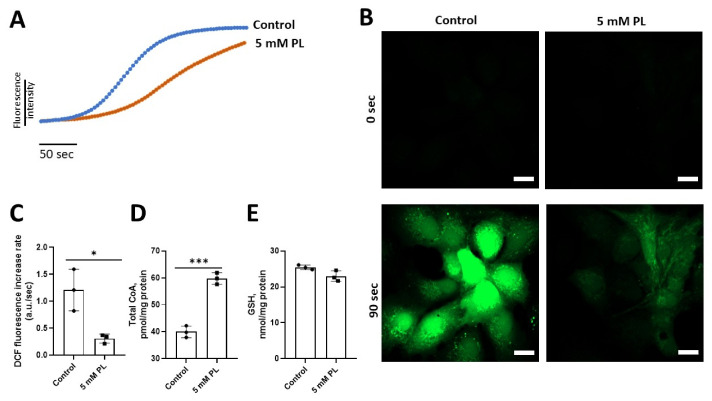
Assessment of the antioxidant activity of panthenol (PL) on the resistance of LX-2 cells to photo-induced oxidative stress during confocal imaging with the ROS-sensitive probe H_2_DCF-DA, as well as measurement of total CoA and GSH levels. LX-2 cells were incubated with 5 mM PL for 24 h. Repeated laser scanning induced photodynamic oxidation of DCF; the kinetics of DCF fluorescence intensity reflected cellular antioxidant capacity under photo-induced ROS production. (**A**) Representative kinetic curves of DCF fluorescence increase during confocal scanning; (**B**) Representative images of DCF fluorescence intensity in LX-2 cells at different time points. Scale bar, 20 μm; (**C**) Rate of DCF fluorescence increase in the linear phase of kinetic curve; (**D**) Intracellular CoA levels; (**E**) Intracellular GSH levels. * *p* < 0.05, *** *p* < 0.001 vs. control (0 mM panthenol) (Mann–Whitney test).

Using confocal microscopy and the ROS-sensitive probe H_2_DCF-DA, we assessed the time-dependent increase in DCF fluorescence in LX-2 cells during repeated laser scanning (Figure 8A,B). Under these conditions, photoexcitation of the probe induces photodynamic ROS generation, which is further detected as progressive DCF fluorescence increases. Therefore, the kinetics of DCF fluorescence intensity in this assay reflect the ability of cells to tolerate photo-induced oxidative stress. Incubation of LX-2 cells with 5 mM PL for 24 h markedly slowed the increase in DCF fluorescence (Figure 8C). The rate of increase in DCF fluorescence at the linear phase of the kinetic curve decreased approximately fourfold, indicating enhanced resistance of LX-2 cells to photo-induced oxidative stress and an increase in the antioxidant capacity upon PL treatment. Importantly, after 24 h, total intracellular CoA levels increased by 33%, whereas GSH levels remained unchanged (Figure 8D,E). Representative time-lapse recordings of DCF fluorescence in LX-2 cells are provided in the Supplementary Materials (Videos S1 and S2).

PL, as an alcohol derivative of pantothenic acid, undergoes enzymatic oxidation by alcohol dehydrogenase (ADH) in liver cells [22]. This reaction enables PL to enter CoA metabolism, which likely leads to an increase in intracellular CoA levels in LX-2 cells. To demonstrate the CoA-mediated mechanism underlying modulation of the antioxidant status of LX-2 cells by PL, we performed a series of experiments using the ADH competitive inhibitor 4-methylpyrazole (4-MP) [23].

**Figure 9:**
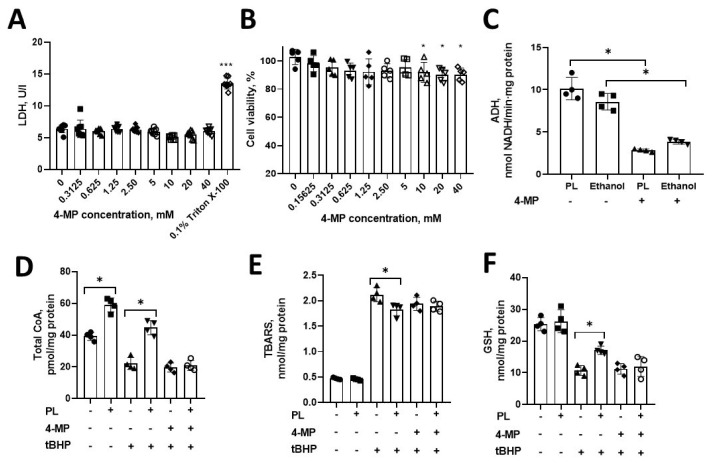
Assessment of CoA biosynthesis and antioxidant status in LX-2 cells treated with PL in the presence of the alcohol dehydrogenase inhibitor 4-methylpyrazole (4-MP) for 24 h, followed by oxidative stress induced by 0.15 mM tBHP for 1 h. (**A**) Cytotoxicity of 4-MP assessed by LDH activity in the culture medium; (**B**) MTT assay following 4-MP treatment; (**C**) Changes in ADH activity in the crude cytosolic fraction of LX-2 cells upon 4-MP treatment; (**D**) Changes in CoA levels following treatment with 4-MP and PL (24 h) and subsequent induction of oxidative stress with tBHP (1 h); (**E**) Changes in TBARS levels following treatment with 4-MP and PL (24 h) and subsequent induction of oxidative stress with tBHP (1 h); (**F**) Changes in GSH levels following treatment with 4-MP and PL (24 h) and subsequent induction of oxidative stress with tBHP (1 h) * *p* < 0.05, *** *p* < 0.001 (Mann–Whitney test).

First, we evaluated the cytotoxic effects of 4-MP over a wide concentration range (Figure 9A,B). LDH activity in the culture medium did not increase upon 4-MP treatment, whereas cell viability of LX-2 cells decreased by approximately 10% at higher concentrations of 4-MP (10–40 mM) (Figure 9A,B).

We further found that treatment with 5 mM 4-MP during 24 h incubation of LX-2 cells resulted in a pronounced (>3-fold) inhibition of ADH activity when PL was used as a substrate, whereas ADH activity was inhibited 2.2-fold when ethanol was used as a substrate (Figure 9C). Importantly, in the absence of 4-MP, PL and ethanol were oxidized by ADH at comparable rates.

During co-incubation of LX-2 cells with 5 mM 4-MP and 5 mM PL for 24 h, differences in cell resistance to oxidative stress induced by 0.15 mM tert-butyl hydroperoxide (tBHP) applied for 1 h at the final stage of the experiment were observed. In control experiments (without 4-MP and tBHP), treatment with 5 mM PL resulted in a 1.5-fold increase in CoA levels, whereas TBARS and GSH levels remained unchanged (Figure 9D–F). Exposure of LX-2 cells to tBHP led to a fourfold increase in TBARS and a twofold decrease in GSH levels.

LX-2 cells pretreated with 5 mM PL were more resistant to tBHP-induced oxidative stress, as evidenced by a 15% decrease in TBARS levels and reduced oxidation of GSH. Notably, co-treatment of LX-2 cells with 5 mM PL and 4-MP did not confer protection against tBHP-induced oxidative stress. Treatment with 4-MP resulted in depletion of CoA levels in LX-2 cells, leading to impairment of the CoA system (but not the GSH system) and increased susceptibility of cells to oxidative damage induced by tBHP.

Taken together, these results demonstrate the involvement of the CoA system, and of PL as its modulator, in the mechanisms underlying resistance of LX-2 cells to oxidative stress.

**Figure 10:**
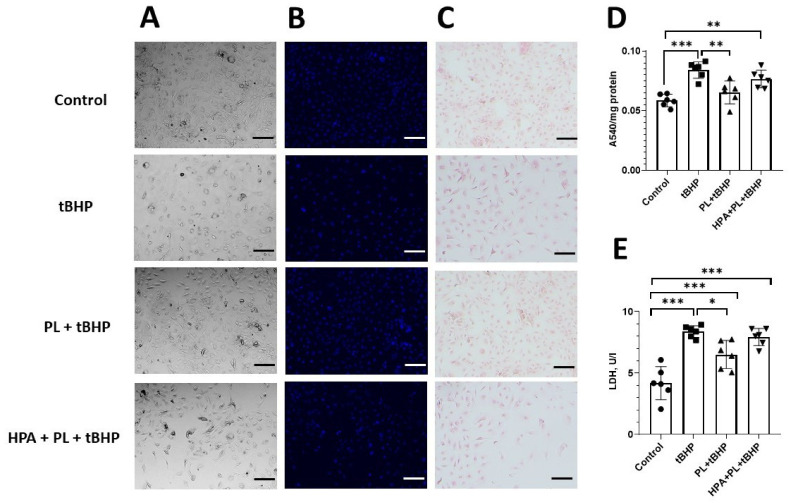
Evaluation of the antifibrotic and cytoprotective effects of PL under oxidative stress induced by tBHP and in the presence of the PANK inhibitor HPA in LX-2 cells. LX-2 cells were cultured with 0.8 mM HPA for 3 days; 5 mM PL was added on day 2, and 0.05 mM tBHP was added for 24 h on day 3 of the experiment. (**A**) Representative light microscopy images of LX-2 cells following 24 h incubation with PL and t-BHP. Scale bar, 150 μm; (**B**) Representative fluorescent images of LX-2 cells stained with DAPI. Scale bar, 100 μm; (**C**) Representative micrographs of LX-2 cells stained with Picrosirius Red. Scale bar, 100 μm; (**D**) Collagen content determined based on the amount of Sirius Red bound and extracted after staining; (**E**) LDH activity in the culture medium. * *p* < 0.05, ** *p* < 0.01, *** *p* < 0.001 (ANOVA, Tukey’s test).

We also evaluated the cytoprotective and antifibrotic activity of PL under oxidative stress induced by tBHP and in the presence of the PANK inhibitor HPA (Figure 10A–E). Oxidative stress induced by tBHP in LX-2 cells resulted in a 30% increase in collagen levels, accompanied by a 50% increase in LDH activity in the culture medium (Figure 10C–E). Treatment with PL under tBHP-induced oxidative stress reduced collagen deposition by 22% and decreased LDH activity in the medium. Importantly, incubation of the cells with HPA prevented the protective effects of PL, suggesting that its protective activity may be associated with the CoA biosynthesis pathway.

**Figure 11:**
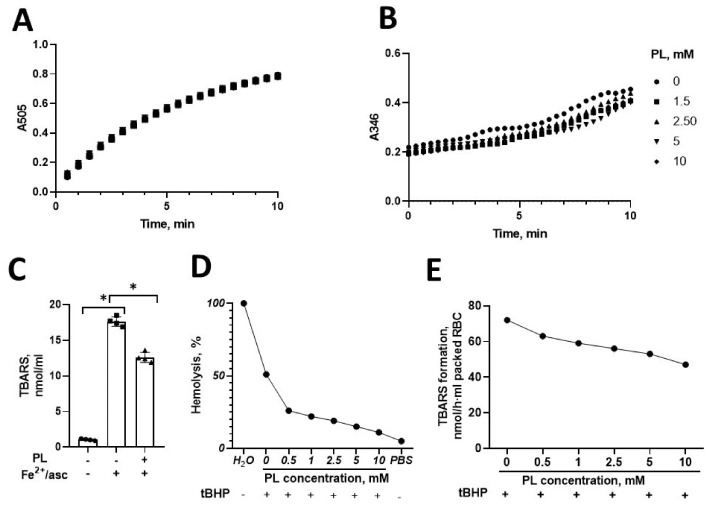
Assessment of the effects of PL on ROS production and membrane resistance to lipid peroxidation in vitro. (**A**) Representative kinetic curves showing the rate of H_2_O_2_ production in the presence of different concentrations of PL; (**B**) Representative kinetic curves showing the rate of superoxide anion production in the presence of different concentrations of PL; (**C**) TBARS levels in azolectin liposomes incubated with 5 mM PL and exposed to 0.05 mM Fe^2+^ and 0.5 mM ascorbate for 1 h; (**D**) Representative curve illustrating the susceptibility of rat RBC membranes to peroxidation induced by 0.7 mM tBHP in the presence of PL for 1 h; (**E**) Representative curve showing changes in the susceptibility of RBC membranes to oxidative hemolysis induced by 0.7 mM t-BHP in the presence of PL for 1 h. * *p* < 0.05 (Mann–Whitney test).

In addition, to investigate a possible alternative mechanism of direct antioxidant action of PL independent of the CoA system, we performed in vitro experiments testing PL in model systems of ROS generation and membrane lipid peroxidation (Figure 11). We found that the addition of PL (1.5–10 mM) to a cell-free enzymatic system for H_2_O_2_ generation (glucose/glucose oxidase system) did not affect its production (Figure 11A). Similarly, PL (1.5–10 mM) did not reduce the rate of superoxide anion production during adrenaline autooxidation (Figure 11B). Thus, PL does not exhibit direct antioxidant activity in vitro.

On the other hand, in membrane lipid peroxidation models, PL demonstrated a protective effect. During Fe^2+^/ascorbate-induced lipid peroxidation of azolectin liposomes, 5 mM PL reduced TBARS levels by 29% (Figure 11C). In a model of oxidative hemolysis induced by 0.7 mM tBHP in a suspension of isolated rat red blood cells (RBC, hematocrit 7%), PL effectively increased resistance to hemolysis and inhibited TBARS formation in a dose-dependent manner with increasing concentrations in the reaction mixture (Figure 11D,E).

Thus, based on the above findings, we suggest that the antifibrotic effects of panthenol in liver tissue and cells are associated with modulation of mitochondrial function, stabilization of CoA levels, and attenuation of oxidative processes, including lipid peroxidation and oxidative protein modification. Further studies are required to elucidate the mechanisms underlying the membrane-protective effects of PL during lipid peroxidation.

## 3. Discussion

The CoA system plays a key role not only in acyl metabolism but also in the antioxidant defense of the cell [9,24]. The novelty of our study lies in an approach aimed at stabilizing the CoA biosynthesis system under oxidative stress conditions in rats subjected to BDL through administration of PL, a xenobiotic precursor of CoA biosynthesis. The use of alternative pharmacological CoA modulators, particularly PT as a native CoA precursor and HPA as an inhibitor of CoA biosynthesis, allowed us to identify the therapeutic potential of PL associated with the CoA system in vivo.

We found that BDL animals exhibited a fourfold decrease in total CoA and a 1.5-fold decrease in free CoA in the liver, accompanied by an increase in protein-bound CoA, indicating a redistribution of CoA between the protein-bound pool and free CoA. This suggests that, under conditions of chronic obstruction, hepatocytes may accumulate CoA in the form of mixed disulfides with proteins, thereby maintaining an optimal redox status for the functioning of antioxidant systems under oxidative stress conditions.

It should be noted that BDL-induced liver injury in rats was associated with decreased CoA levels, likely due to chronic oxidative stress accompanied by impaired mitochondrial functional activity. Importantly, PL administration to BDL rats was accompanied by induction of ADH and PANK, which apparently play a key role in PL metabolism and its incorporation into CoA biosynthesis.

**Figure 12:**
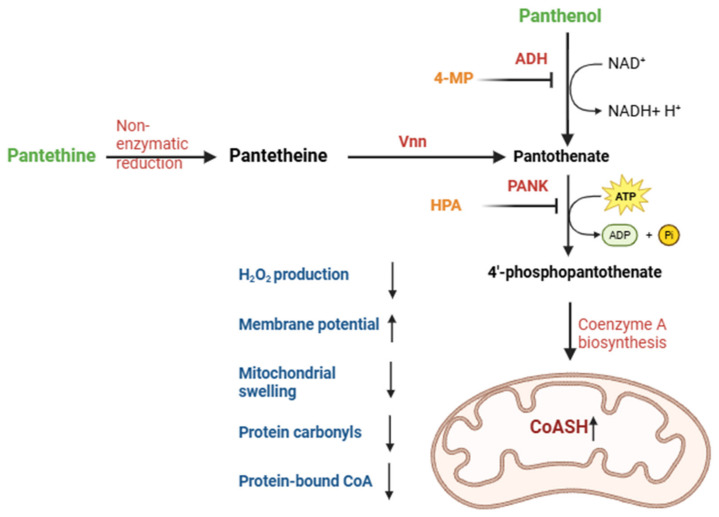
Metabolism and redox-modulating effects of panthenol and pantetheine in cholestatic rat liver. ADH—alcohol dehydrogenase; CoASH—coenzyme A, thiol form; HPA—hopantenic acid (inhibitor PANK); 4-MP—4-methylpyrazole (inhibitor ADH); PANK—pantothenate kinase; Vnn—pantetheinase (vanin). Created in BioRender. Semenovich, D.S. (2026) https://BioRender.com/rxroku0, accessed on 16 February 2026.

Importantly, the biotransformation of PL, as well as that of the alternative CoA biosynthesis modulator PT, is closely linked to their conversion into pantothenate, which enables their incorporation into the CoA biosynthetic pathway (Figure 12). PL, an alcohol derivative of pantothenic acid, serves as a substrate for ADH in hepatocytes, while PT, a disulfide form of pantetheine, acts as a substrate for pantetheinase (vanine, Vnn), both functioning as precursors in CoA biosynthesis [22,25]. In contrast, HPA—a structural homolog of pantothenic acid (N-pantoyl-GABA)—acts as a competitive inhibitor of PANK and thereby suppresses CoA biosynthesis in hepatic cells [18]. It should be noted that HPA administration to BDL rats did not improve the observed histopathological alterations and was accompanied by worsening of redox status parameters and mitochondrial dysfunction. In contrast, PL, to a greater extent than PT, improved metabolic parameters in the liver of BDL rats. Thus, CoA biosynthesis precursors have clear therapeutic potential.

Studies in the human HSC line LX-2 demonstrated that inhibition of ADH by 4-MP abolishes the CoA-mediated protective effects of PL. In addition, modeling of oxidative processes in vitro using cell-free systems of H_2_O_2_ and superoxide radicals revealed that PL does not exhibit direct antioxidant activity. At the same time, in model systems of membrane lipid peroxidation, PL demonstrated pronounced membrane-protective effects, reducing the susceptibility of liposomal and RBC membranes to oxidative damage. Notably, RBCs lack mitochondria, which excludes a CoA-dependent metabolic pathway of PL in this model. These effects may be associated with alterations in the physicochemical properties of membranes, as PL, an aliphatic alcohol, is unlikely to efficiently scavenge ROS. Therefore, the membrane-protective effects of PL require further clarification. The membrane-protective effect of PL and PT, but not HPA, is further supported by parameters of mitochondrial functional activity in BDL rats, including normalization of respiratory activity, stabilization of the mitochondrial membrane potential, and inhibition of Ca^2+^-induced swelling in vitro.

The antifibrotic and indirect antioxidant effects of PL and its analog PT were associated with increased PANK activity and enhanced CoA biosynthesis in the liver of BDL rats. Notably, PL treatment resulted in a marked activation of ADH, which likely plays a key role in its metabolic conversion and subsequent incorporation into the CoA biosynthetic pathway.

Importantly, our data indicate that CoA biosynthesis in cholestatic liver predominantly proceeds via the classical pantothenate-dependent pathway, as *Vnn1* mRNA expression was markedly suppressed and did not recover upon administration of CoA modulators. In addition, hepatic CoA levels may be partially maintained through the release of CoA from protein-bound forms.

Overall, administration of PL and PT as CoA precursors attenuated oxidative stress in BDL rats, as evidenced by reduced mitochondrial H_2_O_2_ production and stabilization of redox balance, leading to partial restoration of mitochondrial function. However, oxidative stress in this model remained pronounced, as reflected by increased protein carbonylation and depletion of GSH, accompanied by reduced levels of free CoA. These changes likely contribute to hepatocyte vulnerability and progression of fibrosis. Importantly, inhibition of CoA biosynthesis in the liver by HPA was associated with increased susceptibility to liver injury and accelerated progression of fibrosis in BDL rats. Thus, increasing the level of CoA in cells can be used along with traditional therapy for liver diseases aimed at normalizing the redox status of mitochondria, which can increase the resistance of hepatocytes to oxidative stress and suppress the progression of fibrosis.

In rats with chronic obstructive cholestasis, characteristic signs of oxidative stress were observed, associated with mitochondrial ROS overproduction and reduced mitochondrial functional activity. Therefore, in the in vitro model using LX-2 hepatic stellate cells, we used tBHP treatment to induce oxidative stress and initiate fibrotic changes.

Under conditions of oxidative stress, free SH-groups of CoA can interact with ROS, along with GSH, forming a pool of mixed disulfides, leading to oxidative reversible modification of protein CoAlation [10]. During protein CoAlation, CoA-SH forms mixed disulfide bonds with reactive cysteine residues of proteins and peptides, thereby protecting important catalytic SH-groups from irreversible oxidative modifications, such as sulfonylation, which may cause structural and functional changes in proteins [14]. Thus, CoAlation contributes to the total antioxidant defense system in liver cells along with the GSH system, whereas the development of oxidative stress leads to excessive oxidation of CoA and formation of mixed disulfides of proteins, which weakens this protective pathway.

There is reason to suggest that direct oxidation of CoA-SH by ROS may lead not only to the formation of CoAlated proteins but also to the generation of symmetric (CoA–S–S–CoA) and mixed disulfides (e.g., with GSH). Thus, the ratio of CoA-SH/CoA–S–S–CoA or CoA–S–S–G may reflect cellular redox status, similarly to the GSH/GSSG ratio. However, disulfide forms of CoA, like those of GSH, represent only a small fraction of the total thiol pool, and their quantification in tissues remains technically challenging. Furthermore, analysis of CoA-modified proteins is currently limited by the lack of specific antibodies for immunochemical detection. It should also be noted that the method of enzymatic assessment of the level of protein-bound CoA detects the sum of mixed disulfide forms of CoA and some amount of conjugated acyl forms of CoA, the contribution of which is difficult to detect. Therefore, it should also be noted that the fractions shown in Figure 4A–C are defined by the extraction procedure. In particular, the protein-bound CoA fraction may contain not only CoA released from CoAlated proteins after reduction but also a certain amount of acyl-CoA species retained during protein precipitation. Accordingly, the sum of free CoA and protein-bound CoA should not be expected to match total CoA quantitatively.

Thus, pharmacological support of cellular CoA homeostasis can be used along with conventional therapy of liver diseases aimed at normalizing bile acid metabolism and reducing inflammation, which in turn can increase the tolerance of hepatocytes to oxidative stress and suppress the progression of fibrosis.

As an alternative to our approach to modulating the CoA system, activators of enzymes involved in its biosynthesis can be investigated. Promising progress has been made in the development of small-molecule PANK activators, particularly PZ-2891, which increases CoA biosynthesis in vivo through allosteric activation of PANK1-3 and demonstrates favorable pharmacokinetics and blood–brain-barrier permeability [26]. Although we did not include PZ-2891 in the current study, such agents represent a mechanistically targeted approach to enhance CoA availability independently of precursor supplementation. Future work should investigate whether pharmacological activation of PANK may provide more robust restoration of hepatic CoA homeostasis and stronger therapeutic benefit in cholestatic liver injury.

Under conditions of chronic cholestasis in rat liver, the GSH pool was markedly depleted, particularly in liver mitochondria, where the activity of key GSH-metabolizing enzymes (GPx and GST) was also reduced due to severe oxidative stress. In the in vitro experiments on HSCs LX-2, the prooxidant effect of tBHP similarly resulted in a twofold decrease in GSH levels, which was not restored upon combined treatment with PL and 4-MP. Treatment with PL alone did not increase GSH levels in HSCs, although it has been reported that PL enhances GSH biosynthesis in human lymphoblast cell cultures [27]. At the same time, it is known that under conditions of GSH depletion induced by APAP (paracetamol) in primary human hepatocyte cultures, 4-MP reduced cell death, exerted a stabilizing effect on GSH levels, and decreased the formation of oxidative products and GSH–APAP conjugates [28]. Overall, we did not anticipate any negative effects of 4-MP and did not specifically investigate its direct effects on HSCs in vitro, as 4-MP is widely used as a safe therapeutic agent (fomepizole) for the prevention of toxic alcohol poisoning in clinical practice [29]. Therefore, we believe that combination therapy in BDL rats using GSH precursors and CoA biosynthesis modulators may be more effective. This hypothesis is partially supported by studies demonstrating the successful use of NAC in combination with PL both in vivo and in vitro in models of oxidative stress [30,31,32]. This approach may be used for antifibrotic therapy and redox balance regulation strategy in chronic liver diseases, which requires further study.

In conclusion, we would like to highlight several important limitations of our study. First, administration of PL and alternative CoA modulators (PT and HPA) was initiated from week 3 to week 6 after BDL. At this stage of obstruction, pronounced metabolic alterations occur in the liver, associated with the development of advanced fibrosis. We assume that the efficacy of PL could be higher if PL administration were started immediately after obstruction, which requires further investigation. Second, in the rat model of BDL-induced chronic liver injury, we did not investigate the combined administration of PL with CoA inhibitors, particularly 4-MP and HPA, which limits the mechanistic interpretation of the hepatoprotective effects of PL under conditions of oxidative stress and inhibition of CoA biosynthesis in vivo.

## 4. Materials and Methods

### 4.1. Animals

Experiments were performed on male Wistar rats aged 7–9 months with a body weight of 430–500 g (n = 25). The study was conducted according to the guidelines of the Declaration of Helsinki and approved by the A.N. Belozersky Institute of Physico-Chemical Biology Lomonosov Moscow State University (protocol 016-1/02/2025 from 9 June 2025). All procedures were executed in accordance with the “Animal Research: Reporting of In Vivo Experiments” (ARRIVE) guidelines. Rats in all experimental groups had unlimited access to drinking water. Throughout the study, rats received pelleted food from Laboratorkorm (Laboratorkorm, Moscow, Russia). Rats were housed under controlled temperature conditions (18–22 °C) with a 12 h light-dark cycle.

### 4.2. Experimental Design

Before the BDL modeling, rats were fasted for 14–16 h with free access to drinking water. Under anesthesia with chloral hydrate (300 mg/kg, intraperitoneally), a midline laparotomy was performed, the liver was accessed, the common bile duct was isolated, and two ligatures were applied with 3-0 silk suture. The common bile duct was divided between the ligatures. In sham-operated rats, the common bile duct was isolated but not ligated or transected. Sutures were applied and covered with 1% brilliant green solution. To prevent postoperative infection, rats were administered the antibiotic (20 mg/kg cefazolin, single dose, intramuscularly). Sutures were removed 10 days after surgery.

Three weeks after surgery, rats with BDL were divided into groups receiving PL or other modulators of coenzyme A biosynthesis (PT and HPA) at an equivalent dose of 500 mg/kg intragastrically daily for 3–6 weeks after obstruction. The PL dose (500 mg/kg) was chosen based on its efficacy in treating acute liver injury in experimental rat models, which is confirmed in these studies [6,33]. It should be noted that, given the molecular weight of the CoA modulators studied, the administered dose corresponds to approximately 2 mmol/kg pantothenic acid equivalents. Sham-operated rats and rats with BDL without treatment were administered an equal volume of distilled water.

Thus, five experimental groups were formed:

1—Sham (n = 5);

2—BDL (n = 5);

3—BDL + PL (n = 5);

4—BDL + PT (n = 5);

5—BDL + HPA (n = 5).

At week 6 of the experiment, rats were euthanized under chloral hydrate anesthesia (300 mg/kg intraperitoneally), and liver tissue samples were then collected for biochemical and morphological analyses.

### 4.3. Isolation of Liver Mitochondrial and Cytosolic Fractions and Total Protein Determination

Mitochondria were collected from rat liver by differential centrifugation [34]. Fresh liver tissue was homogenized in an ice-cold isolation buffer consisting of 0.25 M sucrose, 20 mM Tris-HCl (pH 7.4), 0.05% fatty acid-free BSA, and 1 mM EGTA. A first centrifugation was at 1500× *g* for 10 min at 4 °C, resulting in the formation of a pellet of nuclei and cellular debris. The clear supernatant was then transferred to tubes. The mixture was then centrifuged again at a higher speed at 12,000× *g* for 10 min at 4 °C, yielding a mitochondrial-enriched pellet. To obtain the liver cytosolic fraction, the supernatant was centrifuged in a Beckman ultracentrifuge at 105,000× *g* for 1 h at 4 °C. The mitochondrial pellet was placed in buffered saline solution (150 mM KCl, 20 mM Tris-HCl, pH 7.4) and then diluted to a protein concentration of 40–60 mg/mL. Total protein concentration in this suspension was determined using the modified Lowry method [35].

### 4.4. Assessment of Mitochondrial Functional Parameters

#### 4.4.1. Respiration Activity of Liver Mitochondria

Mitochondrial respiratory activity was assessed polarographically using a Strathkelvin SI782 precision dissolved oxygen respirometer (Cole-Parmer, Vernon Hills, IL, USA). The incubation medium contained 250 mM sucrose, 10 mM MOPS, 2.5 mM MgCl_2_, 1.0 mM EGTA, 2.5 mM KH_2_PO_4_, and 5 mM sodium succinate as a substrate in the presence of 2 μM rotenone, pH 7.2 [36]. The reaction was initiated by adding mitochondria (1 mg/mL total protein). The experiment was carried out at 25 °C. State 2 (V2) corresponds to respiration in the presence of succinate. The mitochondrial respiration rate in state 3 (V_3_) was recorded after the addition of ADP (100 μM). The rate of uncoupled respiration (V_CCCP_) was recorded in the presence of CCCP (0.1 μM). Mitochondrial coupling (respiratory control ratio) was defined as the V_CCCP_/V_2_ ratio. Oxygen consumption curves were obtained and processed using the Strathkelvin Oxygen System. Data Analysis Module software version 3.0.1.16 (Strathkelvin Instruments, North Lanarkshire, Scotland).

#### 4.4.2. Measurement of Mitochondrial H_2_O_2_ Production

H_2_O_2_ production by liver mitochondria was determined by its reaction with amplex red, catalyzed by horseradish peroxidase (HRP), using a kinetic spectrofluorimetric method [37]. The reaction mixture contained 175 mM KCl, 20 mM MOPS, pH 7.2, 1 mM KH_2_PO_4_, 1 mM MgCl_2_, 5 mM sodium succinate, 0.01 mM amplex red, 4 U/mL HRP, and mitochondrial suspension (to 0.05 mg/mL total protein). The increase in fluorescence was recorded at an absorption wavelength of 555 nm and an emission wavelength of 581 nm using a FluoroMax-3 spectrofluorometer (HORIBA Jobin Yvon GmbH, Munich, Germany).

#### 4.4.3. Measurement of Mitochondrial Membrane Potential

Mitochondrial membrane potential was assessed using a kinetic spectrofluorimetric method with safranin O [38]. The incubation medium contained 125 mM sucrose, 50 mM KCl, 20 mM Tris-HCl, 20 mM KH_2_PO_4_, 5 mM MgCl_2_, 0.5 mM EDTA, and 8 μM safranin O. Measurements were initiated by adding mitochondria to a final protein concentration of 0.25 mg/mL. Mitochondria were energized with 5 mM sodium succinate in the presence of 10 μM rotenone. Membrane potential depolarization was induced by the addition of the uncoupler CCCP (2 μM). Measurements were performed at 25 °C. Fluorescence was recorded at an excitation wavelength of 495 nm and an emission wavelength of 586 nm using a FluoroMax-3 spectrofluorometer (HORIBA Jobin Yvon GmbH, Munich, Germany).

#### 4.4.4. Assessment of Ca^2+^-Induced Mitochondrial Swelling

Ca^2+^-induced mitochondrial swelling was measured in a medium containing 250 mM sucrose, 20 mM MOPS, 1 mM KH_2_PO_4_, 5 mM sodium succinate, 1 μM rotenone, pH 7.2 [39]. The mitochondrial suspension was added to the medium before measurement (up to 0.5 mg/mL total protein) and 0.05 mM CaCl_2_ was added. The change in absorbance at 540 nm was recorded on an INNO plate spectrophotometer (LTeck, Seongnam, Republic of Korea) for 15 min at 25 °C. Ca^2+^-induced mitochondrial swelling was measured by the change in light scattering amplitude at 540 nm (ΔA_540_/min).

### 4.5. Assessment of Protein Carbonyl Level, TBARS and SOD Activity

The level of mitochondrial protein carbonyls was determined spectrophotometrically based on their reaction with 2.4-dinitrophenylhydrazine (DNPH) at 366 nm. Protein carbonyl content was expressed as nmol/mg mitochondrial protein using a molar extinction coefficient of 22,000 M^−1^cm^−1^ [40].

The content of TBARS in the cell lysates was determined spectrophotometrically at 532 nm [41]. The level of TBARS was calculated using a molar extinction coefficient of 156,000 M^−1^cm^−1^.

SOD activity was measured in the mitochondrial fraction of rat liver using a spectrophotometric method based on quercetin autooxidation at 405 nm [42]. One unit of SOD activity was defined as the amount of enzyme required to cause 50% inhibition of quercetin autooxidation. Changes in absorbance were recorded in kinetic mode at 25 °C using an INNO microplate spectrophotometer (LTeck, Seongnam, Republic of Korea).

### 4.6. Determination of Reduced Glutathione Levels and Activities of Glutathione-Metabolizing Enzymes and Thioredoxin Reductase

The levels of GSH in liver cytosolic and mitochondrial fractions were determined spectrophotometrically using Ellman’s reagent at 412 nm [43]. Calibration curves were constructed using GSH standards in the concentration range of 0.1–1.0 mM.

The activity of glutathione-metabolizing enzymes was assessed in the mitochondrial fraction of rat liver using kinetic spectrophotometric methods at 340 nm. GPx activity was measured using tBHP as a substrate as described [44]. GPx activity was expressed as the rate of NADPH oxidation during the reduction of oxidized glutathione in a coupled glutathione reductase reaction, using a molar extinction coefficient of 6220 M^−1^·cm^−1^. GST activity was determined using 1-chloro-2,4-dinitrobenzene (CDNB) as a substrate [45]. GST activity was expressed based on the formation of the GSH conjugate with CDNB (GS–CDNB), using a molar extinction coefficient of 9600 M^−1^cm^−1^. Glutathione reductase (GR) activity was determined based on NADPH-dependent reduction of oxidized glutathione, using a molar extinction coefficient of 6220 M^−1^·cm^−1^ [46]. Thioredoxin reductase (TrdR) activity was measured as the rate of NADPH-dependent reduction of 5,5′-dithiobis(2-nitrobenzoic acid) (DTNB) at 405 nm, using a molar extinction coefficient of 14,150 M^−1^·cm^−1^ for 5-thio-2-nitrobenzoic acid (TNB) [47]. Changes in absorbance were recorded in kinetic mode at 37 °C using a Zenyth 3100 multimode microplate reader (Anthos Labtec Instruments, Salzburg, Austria).

### 4.7. Measuring Total, Free and Protein-Bound CoA

To determine total CoA content, liver tissue (20–30 mg) or LX-2 cell pellets were stored in liquid nitrogen and then homogenized in a lysis solution containing 0.25 M KOH in 50% ethanol (*v*/*v*). The resulting homogenate was supplemented with 20 μL of 1 M dithiothreitol (DTT) and incubated for 15 min at 60 °C. After incubation, the samples were plunged into an ice bath. The mixture was then neutralized with 0.5 M triethanolamine hydrochloride prepared in 6% perchloric acid, adjusting the pH to 6–7, and centrifuged at 12,000× *g* for 10 min at 4 °C.

To determine the free CoA content, liver tissue (100 mg) was stored in liquid nitrogen and homogenized in the cold in 0.5 mL of 6% (*w*/*v*) perchloric acid containing 1 mM EDTA. The homogenate was centrifuged, and the supernatant was neutralized as described above and used for analysis.

A recycling enzymatic method with phosphotransacetylase was used to measure CoA [48]. Recycling reagent was prepared, consisting of 60 mM potassium arsenate (pH 7.8), 15 mM acetyl phosphate, and 15 U/mL phosphotransacetylase *Bacillus subtilis* (Megazyme Ltd., Lansing, MI, USA). The reaction was carried out in a 96-well plate, where 15 μL of neutral liver extract was mixed with 30 μL of the recycled reagent and incubated for 24 h at 4 °C. After incubation, the reaction was stopped by adding 5 μL of hydroxylamine hydrochloride (pH 7.0). The samples were incubated at 60 °C for 10 min. Then, 200 μL of the color reagent (8.4% FeCl_3_·6H_2_O in 0.8 M HCl) was added. Absorbance at 540 nm was recorded using an INNO microplate reader (LTeck, Seongnam, Republic of Korea).

For the analysis of protein-bound CoA, a 1:5 (*w*/*v*) liver homogenate was prepared in 6% perchloric acid containing 1 mM EDTA and 20 mM N-ethylmaleimide (NEM). The homogenate was centrifuged, the supernatant was decanted, and the protein precipitate was washed twice with cold acetone. The protein precipitate was solubilized in a solution containing 0.1 M Tris-HCl, 6 M urea, and 5 mM DTT (pH 8.2). Samples were incubated for 30 min, the reaction was stopped by adding 6% perchloric acid, and the samples were centrifuged (12,000× *g*, 10 min, 4 °C). The supernatant was collected, with the pH adjusted to 6–7, and used for the determination of CoA by the recycled method as described above. The content of total, free and protein-bound CoA was determined using a calibration curve in the concentration range of 0.1–2 μM.

### 4.8. Determination of ADH and PANK Activities

The activities of ADH and PANK were measured in the cytosolic fraction of rat liver. A suspension of LX-2 cells (2 × 10^6^ cells/mL) was homogenized with a plastic pestle and centrifuged at 12,000× *g* for 15 min at 4 °C. The resulting supernatant was used as the crude cytosolic fraction for ADH activity measurement.

The reaction mixture for ADH activity (final volume, 220 μL) contained 0.1 M glycine-NaOH buffer (pH 10.0), 1 mM NAD^+^, and 50 mM D-panthenol (or ethanol as a positive control), and 100–150 μg total protein from the cytosolic fraction of liver or LX-2 cells [49]. To confirm the specificity of the reaction with panthenol, 1 mM 4-methylpyrazole (a competitive inhibitor of ADH) was added to the control samples.

The reaction mixture (final volume, 220 μL) for PANK activity consisted of 50 mM Tris-HCl pH 7.4, 5 mM pantothenate, 10 mM MgCl_2_, 5 mM ATP, 5 mM phosphoenolpyruvate, 0.5 mM NADH, 1 U/mL lactate dehydrogenase, and 1 U/mL pyruvate kinase from pig muscles (Reanal, Budapest, Hungary), and 25–50 μg total protein from the cytosolic fraction of liver or LX-2 cells. The reaction was carried out at +37 °C, and the change in absorbance was monitored kinetically for 5 min at 340 nm using a Zenyth 3100 microplate reader (Anthos Labtec Instruments, Wals, Austria). The specific activities of ADH and PANK were calculated using the molar extinction coefficient of NADH (6220 M^−1^cm^−1^) and expressed as nmol NADH/min·mg of total cytosolic protein.

### 4.9. Quantitative Real-Time PCR

*Vnn1* mRNA expression levels were measured using real-time PCR. The 60S ribosomal acidic protein (*Rplp0*) gene was used as a reference for normalization.

Total RNA was isolated from liver tissue using TRIzol reagent (Thermo Fisher Scientific, Carlsbad, CA, USA) according to the manufacturer’s instructions and then processed with DNase I (cat. number EN0525, Thermo Scientific, Waltham, MA, USA, 1000 U/mL). Complementary DNA (cDNA) was obtained using the MMLV RT Kit (Evrogen, Moscow, Russia) strictly according to the manufacturer’s protocol.

Quantitative real-time PCR was performed on a Gentier96E Real-Time PCR System (Xi’an Tianlong Technology Co., Ltd., Xi’an, China) using 5× qPCRmix-HS Master Mix (Evrogen, Moscow, Russia). Primer pairs were designed using the Benchling (https://www.benchling.com) (San Francisco, CA, USA) and Primer-BLAST (https://blast.ncbi.nlm.nih.gov) (NCBI/NLM, Bethesda, MD, USA) cloud services. All oligonucleotides were subsequently synthesized by DNA-synthesis (Moscow, Russia). Primer sequences are presented in Table A1 in Appendix B.

### 4.10. Histological Examination

Histological examination of rat liver tissue was performed to assess structural changes and fibrosis progression. Liver tissue was fixed in 10% buffered formalin and embedded in paraffin using the established method. Paraffin sections (5 µm thick) were stained with Sirius Red using a BioVitrum kit (BioVitrum, St. Petersburg, Russia) according to the manufacturer’s instructions. Stained sections were examined under an AxioScope A1 microscope (Carl Zeiss, Oberkochen, Germany). Stained liver slides were examined at 10× and 20× magnification. Pathological changes in samples from each rat were assessed by examining five to ten selected fields of view. The sections were then photographed.

Histopathological changes in rat liver sections were evaluated using an integrated semi-quantitative scoring system. This assessment included four parameters: inflammation, necrosis, ductular reaction, and fibrosis. Each parameter was assessed on a scale from 0 to 3 according to the criteria specified in Table 2.

The total integrated score of BDL-induced liver injury was calculated as the sum of the scores for all four parameters (ranging from 0 to 12 points for each rat), with higher values indicating more severe histological liver injury. Scoring was performed independently by two pathologists using at least 10 fields of view per rat. The results were expressed as median (min–max) values.

### 4.11. Determination of Hepatic Collagen Content

Total collagen content in liver tissue was assessed based on the determination of 4-hydroxyproline (Hyp). Liver tissue samples (20 mg) were placed in 7 M potassium hydroxide (0.5 mL) and subjected to hydrolysis at 121 °C for 40 min. The resulting hydrolysates were neutralized with 7 M hydrochloric acid and used for spectrophotometric determination of Hyp using Ehrlich’s reagent at 550 nm [50]. Absorbance was measured using an INNO microplate spectrophotometer (LTeck, Seongnam, Republic of Korea). A calibration curve was constructed using Hyp standards. Collagen content was calculated assuming that Hyp constitutes 13.4% (*w*/*w*) of total collagen and expressed as μg/g wet tissue [51].

### 4.12. LX-2 Cell Culture and Assessment of Panthenol Under ADH Inhibition and Oxidative Stress

The experiments were carried out on a cell culture of human liver stellate cells LX-2 (CL-0560, SYNTHBIO, Hefei, China). Cells were cultured in DMEM/F-12 medium (1:1, *v*/*v*) containing 10% fetal bovine serum, 2 mM L-glutamine, 50 U/mL penicillin, and 50 μg/mL streptomycin. All components of the culture medium were purchased from PanEco company (PanEco, Moscow, Russia). The cells were cultured in a CO_2_ incubator at 37 °C in a gas atmosphere containing 5% CO_2_.

Stock solutions of PL (1 M), 4-MP (0.5 M), calcium HPA (0.1 M), and tBHP (0.05 M) were prepared in Hanks’ balanced salt solution without calcium and magnesium salts (PanEco, Moscow, Russia).

For experiments with 5 mM 4-MP and 5 mM PL, LX-2 cells were cultured in T175 culture flasks for 24 h. Oxidative stress was induced by adding 0.15 mM t-BHP for 1 h. The cell monolayer was detached using 0.05% trypsin containing EDTA. Cells were pelleted by centrifugation (300× *g*, 5 min), washed with PBS, and lysed for the determination of CoA, TBARS, and GSH levels.

For experiments with HPA (a PANK inhibitor), LX-2 cells were cultured in 96-well plates (3 × 10^3^ cells per well). HPA (0.8 mM) was added on day 1, followed by panthenol (PL, 5 mM) on day, and 0.05-mM t-BHP on day 3 to induce oxidative stress.

For ADH activity measurements, cell lysates were prepared in 1× PBS pH 7.4, followed by centrifugation (12,000× *g*, 15 min at 4 °C) to remove membrane fractions. The supernatant was used as a crude cytosolic fraction. Total protein content in cell lysates was determined using a modified Lowry method [35].

### 4.13. Assessment of Cytotoxicity and Cell Viability in LX-2 Cells

To evaluate the cytotoxic effects of PL inhibitor ADH 4-MP, LX-2 cells were cultured in 96-well plates. The culture medium was supplemented with PL (0–80 mM) and/or 4-MP (0–40 mM), and the plates were incubated for 24 h in a CO_2_ incubator. A 0.1% Triton X-100 solution was used as a positive control for cytotoxicity. After 24 h, the culture medium was collected to assess cytotoxicity based on lactate dehydrogenase (LDH) release. LDH activity was determined spectrophotometrically at 490 nm as previously described [52].

Cell viability of LX-2 cells was evaluated using the thiazolyl blue tetrazolium bromide (MTT) assay after 24 h of incubation with PL and/or 4-MP. A solution of MTT (2 mg/mL), prepared in sodium bicarbonate-free DMEM/F-12 medium (GE Healthcare, Chicago, IL, USA), was added to each well and incubated for 2 h at 37 °C. The medium was then aspirated, and the formed formazan crystals were dissolved in 100 μL of dimethyl sulfoxide (DMSO). The plates were shaken for 5 min, and absorbance was measured at 570 and 630 nm using an INNO microplate spectrophotometer (LTeck, Seongnam, Republic of Korea). Cell viability was expressed as a percentage relative to control cells not treated with the tested compounds.

To assess the effect of oxidative stress induced by 0.05 mM t-BHP for 24 h in the presence of 5 mM PL on cell number, nuclear staining with 4′,6-diamidino-2-phenylindole (DAPI) (Lumiprobe, Moscow, Russia) was performed to evaluate cell proliferation and cytotoxicity. After 24 h incubation in complete medium with PL and t-BHP, the cell monolayer was washed with 1× PBS pH 7.4 and fixed with 4% formaldehyde in PBS for 15 min at 37 °C. Following fixation, cells were washed three times with PBS (Helicon, Moscow, Russia) to remove residual fixative. Cell nuclei were stained with 300 nM DAPI in 1× PBS pH 7.4 for 30 min at room temperature in the dark. Fluorescence images were acquired using a CELENA^®^ X High Content Imaging System (Logos Biosystems, Anyang-si, Gyeonggi-do, Republic of Korea) equipped with a DAPI filter set (Ex 375/28, Em 460/50). Five replicates were analyzed for each experiment.

### 4.14. Assessment of the Antioxidant Activity of Panthenol (PL) on the Resistance of LX-2 Cells to Photo-Induced Oxidative Stress

Using confocal microscopy and the ROS-sensitive probe H_2_DCF-DA, we assessed the time-dependent increase in DCF fluorescence in LX-2 cells during repeated laser scanning. Under these conditions, photoexcitation of the probe induces photodynamic ROS generation, which is further detected as progressive DCF fluorescence increases.

LX-2 cells were cultured in 50 mm Petri dishes in the presence of 5 mM PL or without PL. After 24 h, the medium was aspirated, and Hanks’ solution containing 10 μM H_2_DCF-DA was added. Cells were incubated at 37 °C for 30 min. 2′,7′-Dichlorofluorescein (DCF) fluorescence was measured using an excitation wavelength of 488 nm and emission collected at 505–550 nm with an LSM 900 confocal microscope (Zeiss, Jena, Germany), provided by the Moscow State University Development Program (Project No. 113). Therefore, the kinetics of DCF fluorescence intensity in this assay reflect the ability of LX-2 cells to tolerate photo-induced oxidative stress. The rate of DCF formation was calculated from the linear portion of the kinetic curve.

### 4.15. Picrosirius Red Staining of LX-2 Cells and Quantification of Total Collagen Content

LX-2 cells were cultured in 6-well plates in the presence of 5 mM PL and 0.05 mM t-BHP for 24 h. The culture medium was then aspirated, and cells were fixed with Bouin’s solution for 1 h at room temperature. Cells were stained with 0.01% Sirius Red in saturated picric acid for 1 h [53]. After staining, cells were washed with 0.01 M hydrochloric acid and imaged. Images were acquired using a CELENA^®^ X High Content Imaging System (Logos Biosystems, Anyang-si, Gyeonggi-do, Republic of Korea). For quantification, the bound dye was extracted with 0.1 M sodium hydroxide, and absorbance was measured at 540 nm using an INNO microplate spectrophotometer (LTeck, Seongnam, Republic of Korea). The amount of dye bound to collagen was normalized to total protein content in the alkaline cell extract.

### 4.16. Assessment of the Antioxidant Activity of Panthenol in In Vitro Models of ROS Generation and Lipid Peroxidation

#### 4.16.1. Assessment of H_2_O_2_-Scavenging Capacity

The hydrogen peroxide-scavenging capacity of PL was assessed in an enzymatic generation system through the oxidation of D-glucose by glucose oxidase. The reaction mixture contained 10 mM D-glucose, 10 mM phenol, 0.15 mM 4-aminoantipyrine, 15 U/mL glucose oxidase (Agat-Med, Moscow, Russia), and 1.5 U/mL horseradish peroxidase (Agat-Med, Moscow, Russia) in 50 mM potassium phosphate buffer, pH 7.5. PL (0–10 mM) was added before adding glucose to the reaction mixture. Changes in absorbance at 505 nm were recorded in kinetic mode for 10 min at 25 °C.

#### 4.16.2. Assessment of Superoxide-Scavenging Capacity

The superoxide-scavenging capacity of PL was evaluated in a non-enzymatic system based on the autoxidation of adrenaline in alkaline medium. The reaction mixture contained 0.05 mg/mL adrenaline hydrochloride (Moscow Endocrine Plant, Moscow, Russia) in 200 mM sodium carbonate buffer (pH 10.6) [54]. PL (0–10 mM) was added to the reaction mixture prior to the addition of adrenaline. Changes in absorbance at 346 nm were recorded in kinetic mode for 10 min at 25 °C.

#### 4.16.3. Assessment of Fe^2+^/Ascorbate-Induced Lipid Peroxidation in Liposomal Membranes

Liposomes were prepared by sonication of an azolectin suspension (10 mg/mL) in 175 mM KCl and 20 mM MOPS (pH 7.2). The reaction mixture contained 175 mM KCl, 20 mM MOPS (pH 7.2), 5 mg/mL azolectin liposomes, 0.05 mM Mohr’s salt, and 0.5 mM ascorbic acid, in the presence or absence of 5 mM PL. Samples were incubated for 1 h at 37 °C. The reaction was terminated by adding thiobarbituric acid (TBA) reagent (0.75% TBA, 17% TCA, *w*/*v*) in a 1:1 (*v*/*v*) ratio, followed by boiling for 15 min. Samples were then cooled on ice, and the colored complex was extracted with 1-butanol (1:1, *v*/*v*). The butanol phase was transferred into 96-well plates (100 μL per well), and absorbance was measured at 532 nm. The concentration of TBARS formation (nmol/mL) was calculated using a calibration curve constructed with 1,1,3,3-tetramethoxypropane (5–50 μM).

#### 4.16.4. Assessment of RBC Membrane Resistance to tBHP-Induced Hemolysis and Lipid Peroxidation

Whole blood was collected from rats into tubes containing sodium heparin and centrifuged at 1200× *g* for 10 min at 4 °C. Plasma and the buffy coat were removed, and red blood cells (RBC) were washed three times with cold 1× PBS, pH 7.4. The hematocrit of the packed RBC suspension was assumed to be 70%.

A 7% hematocrit RBC suspension was prepared in 1× PBS (pH 7.4) containing PL (0–10 mM). For the determination of 100% hemolysis, RBCs were lysed in distilled water. Lipid peroxidation was induced by adding 0.7 mM t-BHP, followed by incubation for 1 h at 37 °C. After incubation, RBCs were pelleted by centrifugation (1200× *g*, 5 min, 4 °C). Hemoglobin release was assessed by measuring absorbance at 540 nm in the supernatant [55]. The level of TBARS was determined as described in Section 4.16.3.

All absorbance measurements were performed using an INNO microplate spectrophotometer (LTeck, Seongnam, Republic of Korea).

### 4.17. Statistical and Bioinformatic Data Analysis

Statistical analysis was performed using GraphPad Prism 8 (GraphPad Software Inc., La Jolla, CA, USA). The data were tested for normality using the Shapiro–Wilk test. Depending on the distribution type, either a parametric one-way analysis of variance (ANOVA) followed by Dunnett’s test, or a nonparametric Mann–Whitney test was applied. Results are presented as the mean ± standard deviation (SD). Differences were considered statistically significant at * *p* < 0.05, ** *p* < 0.01, *** *p* < 0.001 vs. control, or # *p* < 0.05, ## *p* < 0.01, ### *p* < 0.001 vs. BDL group.

For principal component analysis (PCA), the data were analyzed using MetaboAnalyst 5.0. Heatmapper (http://heatmapper.ca/, accessed on 27 March 2026) was used for hierarchical cluster analysis. For the analysis, the mean values of individual parameters were normalized to the mean values in the sham-operated control group, and the common logarithm was performed. The Euclidean distance algorithm and the complete linkage clustering algorithm were selected for heatmap visualization.

## 5. Conclusions

Our study demonstrated the involvement of the CoA biosynthesis system in protecting liver cells in rats with chronic obstructive cholestasis induced by BDL. We found that 6 weeks after BDL, significant alterations were observed in the CoA system. In particular, the activity of PANK and the mRNA expression of vanin-1 (*Vnn1*), key components of CoA biosynthesis from pantothenate, were reduced. This was accompanied by a marked depletion of the free CoA pool in the liver and an increase in protein-bound CoA levels. These findings suggest enhanced oxidation of CoA, along with glutathione, leading to their redistribution between free and protein-bound pools under conditions of persistent oxidative stress and fibrosis.

Therapeutic administration of CoA biosynthesis precursors PL and PT to rats during weeks 3–6 after BDL effectively attenuated oxidative stress and fibrosis in the liver. In contrast, HPA, a competitive inhibitor of PANK, did not exert protective effects, likely due to suppression of CoA biosynthesis, and instead exacerbated liver injury and fibrogenesis. PT showed a somewhat weaker effect than PL but still improved mitochondrial function and oxidative stress parameters, thereby limiting fibrosis progression.

It should be noted that in vitro, PL treatment of hepatic stellate cells was associated with a significant increase in intracellular CoA levels. However, inhibition of ADH by 4-methylpyrazole abolished this effect and increased cellular susceptibility to oxidative stress. At the same time, PL effectively reduced collagen accumulation in stellate cells under oxidative stress conditions.

Importantly, PL does not exhibit direct antioxidant activity in terms of ROS scavenging in vitro. However, PL effectively inhibited lipid peroxidation in model systems of liposomes and RBCs, indicating its membrane-protective properties.

Taken together, the stabilization of the CoA biosynthesis system may contribute to the increased resistance of liver cells to cholestatic injury. CoA biosynthesis precursors, such as panthenol and pantethine, may therefore represent promising hepatoprotective agents for the treatment of chronic cholestatic liver diseases.

## Figures and Tables

**Table 1:** Results of semi-quantitative histopathological assessment of BDL-induced liver injury in rats treated with PL, PT, and HPA from week 3 to week 6 after obstruction. Values are presented as median (min–max).

Groups	Integrated Histopathological Score of Liver Damage (from 0 to 12 Points)
Sham (n = 5)	0 (0–0)
BDL (n = 5)	10 (9–11)
BDL + PL (n = 5)	7 (5–8)
BDL + PT (n = 5)	9 (9–11)
BDL + HPA (n = 5)	10 (10–12)

The integrated histopathological score was calculated as the sum of scores for four parameters: inflammation, necrosis, ductular reaction, and fibrosis. Each parameter was scored from 0 to 3 points. Thus, the integrated score based on the four parameters ranged from 0 to 12 for each rat in the group. Detailed criteria for histopathological scoring are presented in Section 4.10.

**Table 2:** Criteria for semiquantitative histopathological assessment of BDL-induced liver injury in rats.

Scale	Inflammation	Necrosis	Ductular Reaction	Fibrosis
0	None	None	None	None
1	Focal inflammatory infiltrates	Isolated necrotic hepatocytes	Mild bile ductular hyperplasia	Portal fibrosis without septa
2	Multifocal inflammatory infiltrates	Zonal necrosis (centrilobular, periportal)	Moderate bile ductular hyperplasia	Portal fibrosis with rare septa
3	Diffuse inflammatory infiltrates	Localized (multilobular) necrosis	Marked bile ductular hyperplasia with portal/periportal expansion	Portal fibrosis with numerous septa

## Data Availability

The original contributions presented in this study are included in the article and Supplementary Materials. Further inquiries can be directed to the corresponding authors.

## Supplementary Materials

The following supporting information can be downloaded at https://www.mdpi.com/article/10.3390/ijms27114913/s1.

## Abbreviations

The following abbreviations are used in this manuscript:

ADH	Alcohol dehydrogenase
BDL	Bile duct ligation
CCCP	Carbonyl cyanide 3-chlorophenylhydrazone
CoA	Coenzyme A
DCF	2′,7′-Dichlorofluorescein
DMSO	Dimethylsulfoxide
DNPH	2,4-Dinintrophenylhydrazine
EDTA	Ethylenediaminetetraacetic acid disodium salt dihydrate
GSH	Reduced glutathione
GST	Glutathione-S-transferase
GPx	Glutathione peroxidase
GR	Glutathione reductase
H_2_DCF-DA	2′7′-Dichlorodihydrofluorescein diacetate
HPA	Hopantenic acid
Hyp	4-Hydroxyproline
LDH	Lactate dehydrogenase
4-MP	4-Methylpyrazole
MTT	Thiazolyl blue tetrazolium bromide
PANK	Pantothenate kinase
PBS	Phosphate-buffered saline
PL	Panthenol
PT	Pantethine
RBC	Red blood cell
ROS	Reactive oxygen species
SOD	Superoxide dismutase
TBARS	Thiobarbituric acid reactive substances
tBHP	tert-Butyl hydroperoxide
Vnn1	Vanin (pantetheinase)

## Appendix B

**Table A1:** Primer sequences used to assess mRNA expression of the *Vnn1* and *Rplp0* genes.

Protein	Gene Name	Primer Nucleotide Sequence (5′-to-3′)	PCR Product Size, bp	Genbank Accession Number
Vanin 1	*Vnn1*	forward: TATGTCTTCCCTGAGGTGTTGC reverse: TGCCCAGTCCTTCTCATACAAC	153	NM_001025623.1
60S acidic ribosomal protein P0	*Rplp0*	forward: CACAGTACCTGCTCAGAACAC reverse: ACCTTGTCTCCAGTCTTTATCAG	138	NM_022402.2
